# VoDEx: a Python library for time annotation and management of volumetric functional imaging data

**DOI:** 10.1093/bioinformatics/btad568

**Published:** 2023-09-12

**Authors:** Anna Nadtochiy, Peter Luu, Scott E Fraser, Thai V Truong

**Affiliations:** Department of Quantitative and Computational Biology, University of Southern California, Los Angeles, CA 90089, United States; Translational Imaging Center, University of Southern California, Los Angeles, CA 90089, United States; Translational Imaging Center, University of Southern California, Los Angeles, CA 90089, United States; Division of Molecular and Computational Biology, Department of Biological Sciences, University of Southern California, Los Angeles, CA 90089, United States; Department of Quantitative and Computational Biology, University of Southern California, Los Angeles, CA 90089, United States; Translational Imaging Center, University of Southern California, Los Angeles, CA 90089, United States; Division of Molecular and Computational Biology, Department of Biological Sciences, University of Southern California, Los Angeles, CA 90089, United States; Translational Imaging Center, University of Southern California, Los Angeles, CA 90089, United States; Division of Molecular and Computational Biology, Department of Biological Sciences, University of Southern California, Los Angeles, CA 90089, United States

## Abstract

**Summary:**

In functional imaging studies, accurately synchronizing the time course of experimental manipulations and stimulus presentations with resulting imaging data is crucial for analysis. Current software tools lack such functionality, requiring manual processing of the experimental and imaging data, which is error-prone and potentially non-reproducible. We present VoDEx, an open-source Python library that streamlines the data management and analysis of functional imaging data. VoDEx synchronizes the experimental timeline and events (e.g. presented stimuli, recorded behavior) with imaging data. VoDEx provides tools for logging and storing the timeline annotation, and enables retrieval of imaging data based on specific time-based and manipulation-based experimental conditions.

**Availability and implementation:**

VoDEx is an open-source Python library and can be installed via the “pip install” command. It is released under a BSD license, and its source code is publicly accessible on GitHub (https://github.com/LemonJust/vodex). A graphical interface is available as a napari-vodex plugin, which can be installed through the napari plugins menu or using “pip install.” The source code for the napari plugin is available on GitHub (https://github.com/LemonJust/napari-vodex). The software version at the time of submission is archived at Zenodo (version v1.0.18, https://zenodo.org/record/8061531).

## 1 Introduction

Volumetric functional imaging is widely used in neuroscience studies for recording brain activity in parallel with behavioral and physiological data of organisms (Kim and Schnitzer 2022). Such studies often have complex experimental designs, aiming to characterize neural responses to experimental tasks and/or detect differences in brain activity patterns among various stimuli, behaviors, and physiological states.

Accurate analysis of functional imaging data requires accurate annotations of the time course of the experiment and synchronization of the time annotations with the imaging data. Although both commercial and open-source tools are available for designing experiments and tracking behavior (Mathis *et al.* 2018, Peirce *et al.* 2019, Lopes and Monteiro 2021, Akam *et al.* 2022, Gabriel *et al.* 2022), they lack the ability to directly link this information to the imaging data.

Emerging standardized formats like Neurodata Without Borders (Rübel *et al.* 2019) and Brain Imaging Data Structure (Gorgolewski *et al.* 2016) allow for the storage of annotations with imaging data and feature tools for comprehensive processing of these annotations. However, their complexity and time-consuming implementation can create barriers for some users and use cases.

As a result, processing and time-annotating data are often performed manually. Manual data management and processing is not scalable, is hard to document, and is prone to human error, risking potential mistakes that are difficult to detect and correct.

Linking time annotations to volumetric imaging data presents an additional challenge, as volumes are acquired as a series of optical sections: a series of 2D images are taken sequentially at different depths inside the sample, which are then assembled into a 3D dataset. In order to correctly interpret the volumetric data, it is crucial to track not only the correspondence between image frames and experimental conditions, but also track the exact location of these frames within a volume. Such complexity of time annotation, combined with the unprecedented amount of data produced by functional imaging experiments, makes manual data handling tedious and unreliable.

To address these challenges, we introduce VoDEx, Volumetric Data and Experiment manager, an open-source Python library for synchronizing time annotations and volumetric information with imaging data. VoDEx is distributed under a BSD 3-clause License, making it freely accessible for academic, commercial, and personal use. VoDEx integrates the information about individual image frames, volumes, and experimental conditions and allows the retrieval of sub-portions of the 3D-time series datasets based on any of these identifiers without the need to load the entire dataset into memory. It logs all information related to the experiment into an SQLite database, enabling later data verification and sharing in accordance with the FAIR (Findable, Accessible, Interoperable, and Reusable) principles (Wilkinson *et al.* 2016, Dempsey *et al.* 2022). VoDEx is not intended to replace the emerging standardized, comprehensive data formats (Gorgolewski *et al.* 2016, Rübel *et al.* 2019). Instead, we designed VoDEx as a complimentary, low-entry-barrier tool to store annotations, file, and volume-related information in a simple, accessible way. This information can be readily converted to any standard-required format, allowing users to choose when and if to adopt more sophisticated data standards. VoDEx is implemented both as a napari (Sofroniew *et al.* 2022) plugin for interactive use with a GUI and as an open-source Python package. Python’s rich ecosystem of libraries, such as NumPy (Harris *et al.* 2020), SciPy (Virtanen *et al.* 2020), and scikit-image (Van der Walt *et al.* 2014), makes VoDEx a useful tool for image analysis and allows for integration into a wide range of analysis pipelines.

## 2 Implementation

VoDEx contains classes that assist in the creation, organization, and storage of information related to image acquisition and time annotation, allowing for the search and retrieval of image data based on specific conditions. VoDEx’s main functionality is split into creating and querying annotations and data loading.

VoDEx provides methods for constructing, validating, and storing time annotations in an SQLite database and abstracts the SQL calls, providing an easy-to-use interface to query the database. VoDEx keeps track of cycle iterations for cyclic events, which is important in behavioral experiments where the subject might become habituated to the repeated stimulus or learn over the course of the experiment. VoDEx contains classes designed to load image data from specific file types, with current support for TIFF, and extendable for other file formats. The package has undergone rigorous testing, achieving 100% test coverage through the use of pytest.

## 3 Usage

**Figure 1.**
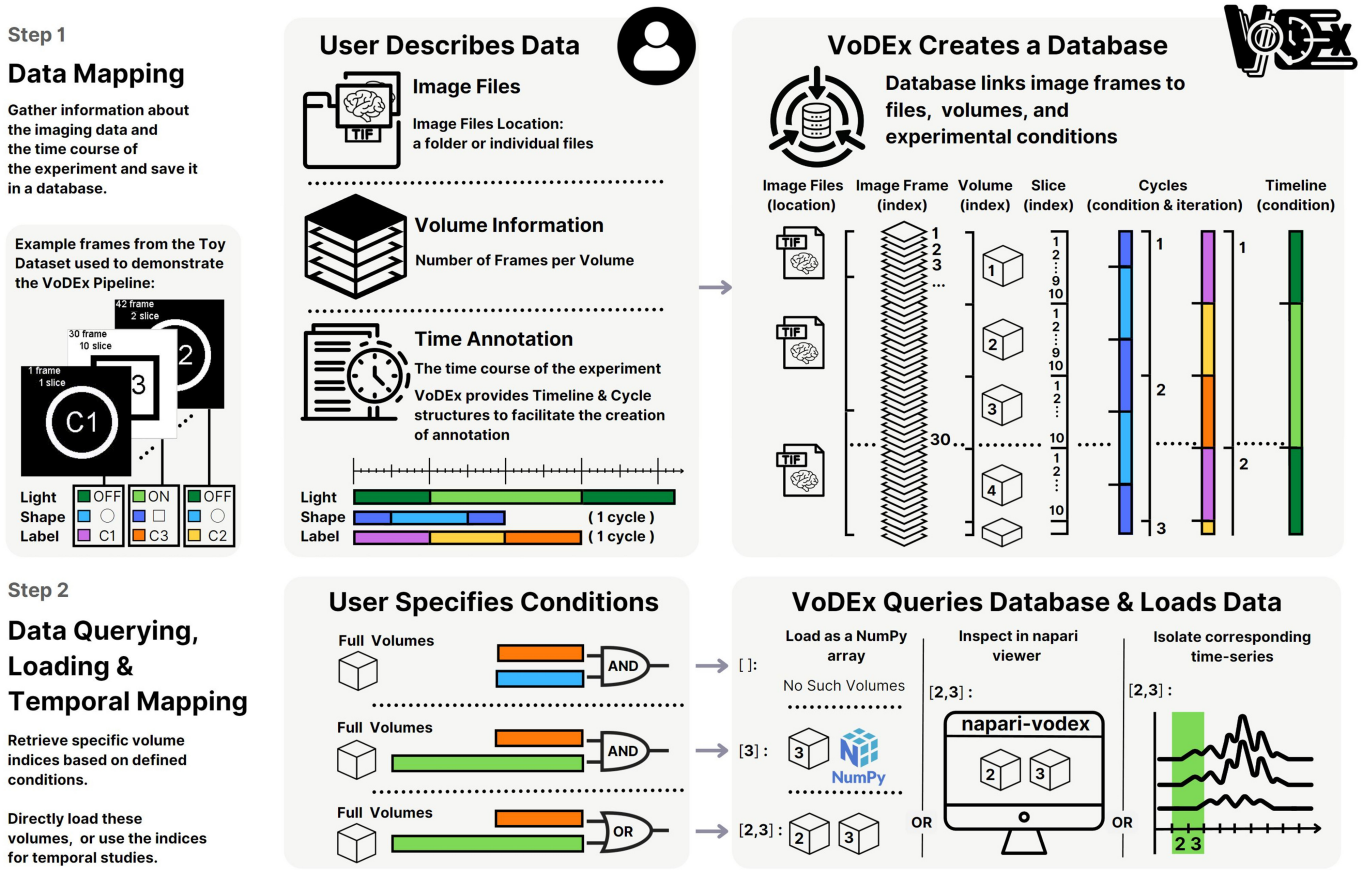
Illustration of the VoDEx pipeline applied to a Toy Dataset. The Toy Dataset is available via the project website at https://lemonjust.github.io/vodex/data/. The dataset comprises 42 image frames, divided into three TIFF files. Each volume consists of 10 frames, resulting in 4 full volumes, with 2 additional frames at the end of the recording. In the dataset, three conditions are tracked: light, label, and shape. The background of the frame indicates the light (on/off), the middle of the screen shows the label (c1, c2, c3), and the screen displays either a circle or a square. In Step 1, the user inputs information about the imaging data and the experimental conditions into VoDEx, either through a Python script or a GUI in the napari plugin. Specifically, the user provides the information for the whole recording to encode light, but only one cycle iteration of the label and shape. Note that the shape conditions switch in the middle of a volume. VoDEx then integrates this information, automatically determining the number of frames in each image file, estimating which frames correspond to which volumes, and repeating the provided cycles to cover the duration of the entire recording while keeping track of both conditions and cycle iterations. The information is stored in a database created by VoDEx. For instance, frame number 30 is stored as a 4th frame inside the 3rd TIFF file. It represents the last slice (10th slice) in the 3rd volume. This frame features a square shape, with the label “c3” and the light “on”. It was recorded during the second iteration of the shape cycle and the first iteration of the label cycle. In Step 2, the user can search and access imaging data based on mapped experimental conditions using either a Python script or the GUI in the napari plugin. Conditions can be combined using “and” or “or” logic. For instance, to retrieve the volumes where the label is c3 and the light is on, VoDEx points to volume index 3. When searching for the label c3 or the light is on, it returns indices for both volumes 2 and 3. If conditions do not span an entire volume, one can pinpoint individual image frames that align with the condition (not depicted). Once the frame or volume indices are determined, VoDEx can load the volumes as numpy arrays for in-depth Python processing or show them directly in the napari viewer. Alternatively, the volume or frame indices can serve as discrete time markers for temporal analysis. This empowers researchers to isolate pertinent intervals from associated time series, such as extracted neural activity.

The VoDEx pipeline consists of two steps: data mapping and data querying. An illustration of the VoDEx pipeline applied to a Toy Dataset is shown in Fig. 1. During the mapping step, VoDEx creates a mapping between image frames, their location within associated files, the image volumes they correspond to, experimental conditions, and cycle iterations. This information is saved to a database, allowing users to save the experiment description for sharing or to return to it later. In the second step, users can investigate the data by querying the database for image frames or volumes that were recorded during specific combinations of the mapped conditions. The querying process does not require prior knowledge of database query syntax and is conducted via methods provided by VoDEx. When requesting frames, VoDEx returns the indices of all the image frames matching the request. When requesting volumes, VoDEx selects those frames that constitute full volumes and returns the corresponding volume indices. The indexing of frames and volumes start from the beginning of the recording. The image data can then be loaded based on these indices as a 3D or a 4D numpy array for frames or volumes, respectively. Alternatively, the volume or frame indices returned by VoDEx can be used as discrete time points to isolate intervals of interest from associated time series, such as extracted neural activity. Typically, each captured volume represents a distinct snapshot of neural activity. This allows VoDEx to isolate specific time intervals that align with particular experimental conditions, effectively using the volumes as discrete time points.

The package offers a graphical user interface (GUI) through its integration with napari via the napari-vodex plugin, enabling users to easily navigate the full VoDEx pipeline and to load selected volumes directly into the napari viewer. The two steps of the pipeline can be performed independently using either the script or the GUI.

Comprehensive documentation of VoDEx, including examples, tutorials, and toy datasets, are provided at the project website https://lemonjust.github.io/vodex/.

## 4 Application

VoDEx is a versatile tool that can be applied to any functional volumetric data. To give concrete application examples, this section focuses on its role in two different methods typically used in processing calcium imaging data.

The first approach, common in high-throughput assays, uses raw imaging data directly and aims to identify large-scale differences in brain activity under various conditions. This type of analysis can be used in drug screening experiments (Lin *et al.* 2018). VoDEx is an ideal tool for data handling in such experiments, as it allows for easy loading and comparisons of image volumes or slices from different conditions.

The second approach starts by extracting activity traces of individual neurons from the imaging data, usually with tools like CaImAn (Giovannucci *et al.* 2019) or Suite2p (Pachitariu *et al.* 2016). The focus then shifts to these activity traces rather than the raw data. VoDEx simplifies this process by streamlining data preprocessing, helping to adjust 3D data for 2D tools, and pinpointing parts of the extracted time series linked to different experimental conditions. While highly effective for 2D data, CaImAn and Suite2p are not directly suitable for large 3D datasets. A common workaround is to use tools like SimpleITK (Beare *et al.* 2018) or ANTs (Avants *et al.* 2011) for 3D motion correction and then process individual image slices with these 2D tools. The same neuron might appear on adjacent slices, thus detected cells from different slices are merged based on activity and location, and, finally, the cell signals are extracted. This method can be complex and time-consuming. VoDEx eases this by offering batch processing, simplifying the 3D motion correction setup. Moreover, its ability to access individual slices from a full recording helps bridge 2D and 3D analysis. After extracting the neural signals, VoDEx can isolate signal parts tied to certain experimental conditions, or aid in building the regressor for activity prediction, when using regression analyses on the signals. Lastly, annotations made with VoDEx can be directly shared as a compact database file for VoDEx users or exported as a CSV file for wider accessibility.

VoDEx is particularly useful for experiments where the time annotation is derived from experimentally measured events, such as an organism’s behavior or physiological data. Unlike well-controlled stimuli sequences that align with the rate of volumetric image collection, transitions between behaviors in such experiments can occur at various times during the recording of individual volumes. VoDEx provides an easy way to detect and manage these types of events.

To demonstrate the capabilities of VoDEx, we present its application to the study of numerosity in zebrafish larvae, where it plays a key role in processing whole-brain calcium imaging data acquired using light-sheet fluorescence microscopy (Supplementary Note).

## 5 Conclusion

VoDEx is an open-source Python library that streamlines the management and analysis of volumetric functional imaging data. The library offers a suite of tools for creating, organizing, and storing information pertaining to image acquisition and time annotation. Additionally, it allows for the retrieval of image data based on specific experimental conditions, enabling researchers to access and analyze the data easily. VoDEx provides a user-friendly solution for processing volumetric functional imaging data, even for researchers without extensive programming experience. VoDEx complements existing standardized formats by simplifying the annotation process, paving the way for adopting more comprehensive formats. The library is designed to be flexible and easily integrated with other Python packages. We envision VoDEx will join the growing list of modular software tools that promote efficient data management and analysis in neuroscientific studies, enabling the community to move toward more widespread sharing and developing of open-source tools.

## Supplementary Material

btad568_Supplementary_Data

## Data Availability

No new data were generated in support of this research.
