# Supplementary material for: VoDEx: a Python library for time annotation and management of volumetric functional imaging data
Source: Bioinformatics. 2023 Sep 12;39(9):btad568. doi: 10.1093/bioinformatics/btad568 (PMC10562951; doi:10.1093/bioinformatics/btad568)
Supplement: btad568_Supplementary_Data [file btad568_supplementary_data.pdf]

Supplementary Note
Zebrafish Numerosity Project: Case-Study for Application of VoDEx

Anna Nadtochiy[1,3,*] Peter Luu[2,3] Scott E. Fraser[1,2,3] and Thai V. Truong[2,3,*]

[1]Department of Quantitative and Computational Biology, University of Southern California, Los Angeles, CA, 90089, USA, [2]Department of Biological Sciences, Division of Molecular and Computational Biology, University of Southern California, Los Angeles, CA, 90089, USA and [3]Translational Imaging Center, University of Southern California, Los Angeles, CA, 90089, USA

*Corresponding authors. nadtochi@usc.edu, tvtruong@usc.edu

1. Numerosity Study

To demonstrate the capabilities of VoDEx, we present its application to the study of numerosity in zebrafish larvae, where it played a key role in the processing of whole-brain functional imaging data acquired using light-sheet fluorescence microscopy (Supplementary Note). Zebrafish larvae were presented with a series of visual stimuli in a pseudo-random order with variable timing. The numerosity stimuli included a blank screen and screens with one to five dots in varying geometric patterns, which are commonly used to control for potential confounding effects (Piazza et al., 2004; Gebuis et al., 2016; Pennock et al., 2021).VoDEx facilitated the interpretation of the responses to the stimuli, which required accurate annotation and tracking of various stimulus patterns. VoDEx efficiently managed the sets of visual stimulus patterns and enabled the processing of large volumetric imaging datasets on a standard computer. The implementation was carried out in Jupyter notebooks and in a custom Python package specifically designed for this study, showcasing the versatility of integrating VoDEx into a comprehensive analysis pipeline. Our preliminary results can be found in Messina et al. (2022).

1.1. Motivation

Numerosity, the ability to perceive and evaluate discrete quantities in a set, is a fundamental aspect of cognition that underpins complex behaviors and decision-making processes in humans and many other species (Nieder, 2020). The zebrafish larva, with its small size, genetic accessibility, and optical transparency, provides an ideal model to study the neural basis for numerosity at the cellular level (Messina et al., 2022).

Previous research identified a region of the zebrafish pallium that selectively responds to changes in the numerosity of visual stimuli (Messina et al., 2022), suggesting an evolutionarily conserved mechanism for approximate numerical magnitude estimation. However, that study focused on a subregion of the pallium, leaving the role of other brain regions in numerosity processing largely unexplored. The study presented in this supplementary note aims to address this knowledge gap by investigating the neural activity throughout the brain of larval zebrafish while they are presented with visual numerosity stimuli consisting of different numbers of dots in various geometric patterns. We describe below how VoDex is used in the data management and analysis of this numerosity study. Complete results from this on-going project will be presented elsewhere.

1.2. Overview of the Experiment and Data Acquisition

To illustrate the application of VoDEx in analyzing the neural activity recorded during the presentation of the visual numerosity stimuli, we will use an individual dataset as an example. The neural activity, reported by the genetically encoded calcium indicator H2B::jGCaMP7f[1] (Dana et al., 2019), was captured using light-sheet microscopy (Keomanee-Dizon et al., 2020) at single-cell resolution. Each brain volume was captured as a sequence of frames and contained 67 frames per volume. This dataset was collected during a numerosity stimuli sequence in which the fish was presented with numerosities 1, 2, 3, 4, and 5. The recording comprises a total of 1900 brain volumes acquired over a period of approximately 2 hours, organized into 17 TIFF files by the acquisition software (Edelstein et al., 2014), with each file having a size of about 4Gb.

1.3. Visual Numerosity Estimation

During the numerosity stimuli presentation, zebrafish larvae were exposed to visual stimuli that aim to isolate neural responses specific to number processing by controlling for both numerical and non-numerical variables. The stimuli consisted of black dots presented on a red background, with the quantity of dots varying from one to five. To account for non-numerical variables that may co-vary with

[1] Transgenic zebrafish provided by Misha Ahrens, Janelia Research Campus.

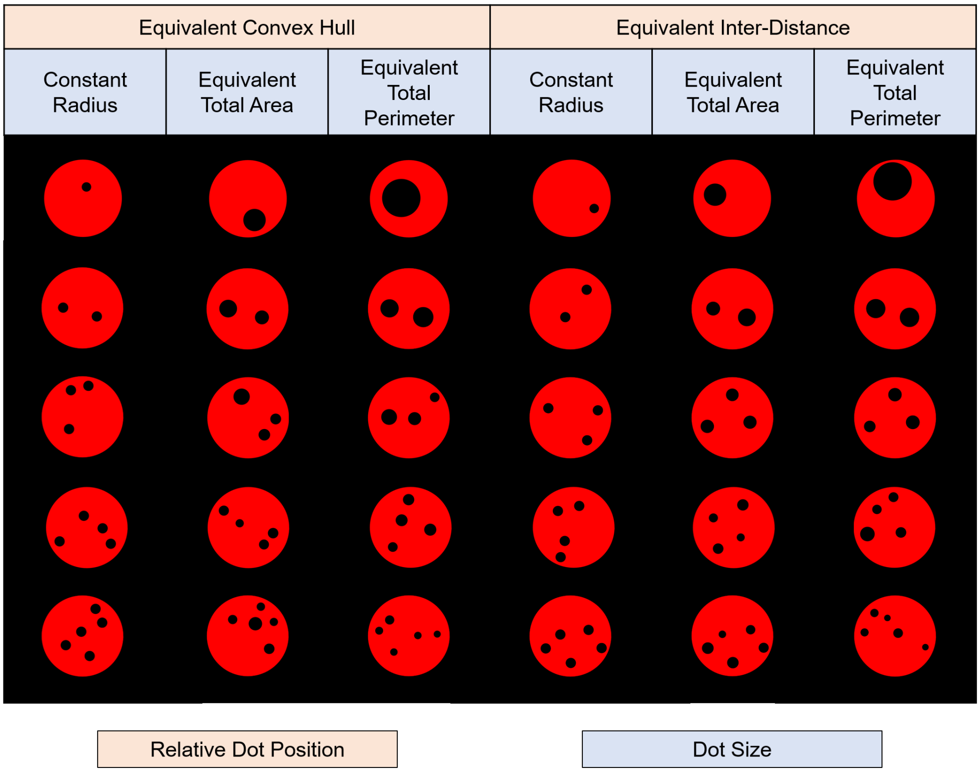

Fig. S1: Figure S1: Visual stimuli used in the numerosity stimuli presentation. The figure illustrates the different geometric parameters used to generate the stimuli across different numerosities. The stimuli maintain either a constant convex hull (smallest convex polygon that encloses all of the elements) or inter-distance (average distance between the dots), while the size and circumference of the dots are controlled by maintaining a constant radius, total area, or total perimeter. Convex hull or inter-distance have no impact on numerosity "1", while convex hull is not taken into account for numerosity "2". A "blank" screen, consisting of the same red background but without any dot pattern, is used to reset between different numerosity stimuli. The patterns were generated using GeNEsIS (Zanon et al., 2021).

the number of dots, two specific geometric parameters were controlled: the relative position of the dots and the size of individual dots. The relative position was regulated by maintaining either an equivalent convex hull or an equivalent inter-distance, ensuring consistent spatial relationships between the dots. The size of the dots was maintained by controlling the dot radius, total dot area, or total dot perimeter for each numerosity (FigS1).

Due to geometric constraints, it was not possible to control for all non-numerical variables simultaneously. Therefore, a pseudo-random stimuli sequence was used (FigS2), which included all possible combinations of relative dot positions and individual dot sizes while varying numerosities. The sequence consisted of 30 different visual stimuli based on combinations of parameters: 2 possible relative positions, 3 possible dot sizes, and 5 numerosities. Such design of the visual stimuli allowed us to isolate the neural responses specific to numerosity and ensure that these responses were independent of variations in other visual cues.

1.4. Data Processing and Analysis of a Single Fish Sample Overview

The data processing pipeline for a single fish sample involved several key steps:

- Firstly, the raw calcium movie was processed to remove slow changes in the calcium signal using a sliding window approach to calculating a relative change in fluorescence (dF/F) per voxel.
- Next, a Statistical Parametric Map (SPM) was created by calculating the difference between brain activity during stimulus presentation and the blank screen (no stimuli) period for each voxel. This SPM highlighted the voxels in the brain images that exhibited significant responses to the stimuli.
- To identify individual cells, adjacent voxels with similar scores in the SPM were grouped together, with each group roughly set to the size of a single cell. This grouping step enabled the extraction of cell signals from the raw movie. To ensure consistency and comparability across cells, the extracted cell signals were further normalized and slow changes in the calcium signal were removed, similar to the processing of raw calcium movies.
- Finally, the cell responses to different numerosity stimuli and different non-numerical variables were compared to assess the significance of differences in cell responses to different visual stimuli. This allowed us to identify the cells that were responsive to a particular numerosity regardless of the dot positions and individual dot sizes.

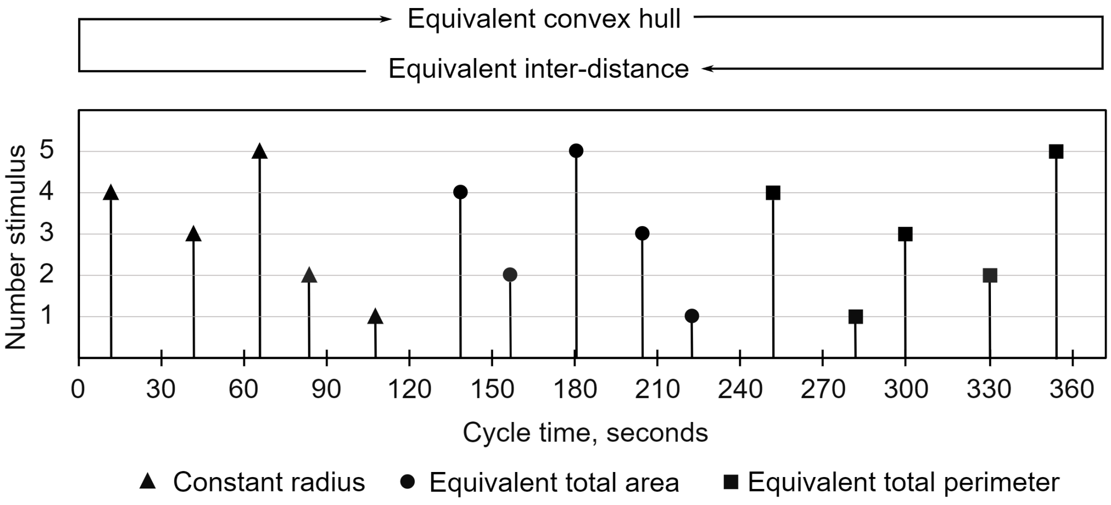

Fig. S2: Figure S2. Pseudo-random stimulus cycle. Each stimulus consists of a 3-second presentation of the dot pattern, followed by a blank screen lasting between 15 and 27 seconds. One cycle has a total duration of 372 seconds. The cycles alternate between two conditions: one with an equivalent convex hull, and the other with an equivalent inter-distance. Each condition includes 15 stimuli, covering all numerosities while maintaining a constant radius, total area, and total perimeter. Different cycles feature distinct dot patterns while adhering to the respective geometric constraints. The cycle is repeated 18 times per sample.

VoDEx proved to be a valuable tool throughout this data processing and analysis pipeline.

2. VoDEx utility for data processing

In section 2.1, we demonstrate the application of VoDEx for batch processing. VoDEx provides an intuitive interface for writing batch-processing scripts that directly operate on brain volumes. This feature simplifies the management and processing of large datasets, such as the numerosity dataset used in this study, which is approximately 60 GB in size. We used batch processing to normalize the raw calcium movies and extract cell signals from them.

Accurate analysis of our numerosity dataset, encompassing 1900 brain volumes and 30 distinct conditions, relies on precise annotation and tracking of stimulus patterns. By utilizing the time annotation and querying capabilities of VoDEx, we ensure the accurate interpretation of neural responses and overcome the challenges associated with the complexity of our stimuli.

In Section 2.2, we highlight the practical applications of VoDEx in selectively loading relevant imaging data, streamlining the creation of the Statistical Parametric Map (SPM).

Section 2.3 expands the scope of VoDEx beyond imaging data and showcases its ability to track complex visual stimuli for the evaluation of the extracted cell responses. With VoDEx, we effortlessly monitor both numerical and non-numerical variables of the presented visual stimuli, enabling precise analysis of the cell signal associated with any specific combination of these variables. This allows us to disentangle the neural responses specific to numerosity from those related to other geometric parameters.

All code examples are using VoDEx version 1.0.18, available at pypi.org/project/vodex/1.0.18/.

2.1. Batch Processing of Calcium Movies

To begin processing the calcium movie, we need to initialize VoDEx by providing the file locations and the number of frames per volume. At this stage, the time annotation information is not necessary and can be included now or added later. In Section 2.3, we will add the time annotation. The following code snippet demonstrates one way of initializing VoDEx:

```
import vodex as vx

# Specify the directory path to the raw data files
data_dir = "path/to/numerosity/raw_data/"

# Specify the number of frames per volume and the starting slice
frames_per_volume = 67
starting_slice = 0

```

```
# Create the experiment object from the directory using the specified parameters
experiment = vx.Experiment.from_dir(data_dir, frames_per_volume, starting_slice, verbose=True)

# Save the experiment for future use
experiment.save("experiment.db")
```

For more detailed information and alternative methods to initialize VoDEx, please refer to the documentation or the quick start guide available at https://lemonjust.github.io/vodex/qstart/new_experiment/.

Once VoDEx is initialized, the experiment object can be used for batch-processing of the dataset. All the necessary methods, including splitting the full brain volumes into batches and loading the volumes, are provided by VoDEx. A template for a batch-processing code is shown below:

```
# Split the volumes into chunks of the defined size that will be loaded into RAM at once
chunks = experiment.batch_volumes(
            batch_size, # the number of volumes in each batch
            full_only=True, # if True, only full volumes are returned
            overlap=overlap # the number of volumes that overlap between batches
                                )
for chunk in chunks:
        # Load the full volumes in the chunk
        data = experiment.load_volumes(chunk, verbose=False)
        # Perform processing
        # ...
```

For further implementation details and usage of batch processing in our pipeline, please refer to the online repository at https://github.com/LemonJust/numan/blob/main/src/numan/analysis.py. The Preprocess class's batch_dff method and the Signals class's from_spots method can be found there, demonstrating the normalization of raw calcium movies and the extraction of cell signals, respectively.

The full preprocessing step, including batch processing, can be found in the Jupyter notebook 01_Define_experiment_and_create_dff_movie.ipynb [2]. Additionally, the signal extraction step is part of the notebook 03_Extract_cell_signals.ipynb [3].

2.2. Streamlining Data Loading for SPM Creation using VoDEx

Here, we describe how VoDEx is used to streamline the data loading process for the creation of the Statistical Parametric Map (SPM).

To start the analysis of neural responses to visual stimuli, we incorporate time annotation into VoDEx. At this stage of the analysis, we only need to distinguish between stimuli and no stimuli conditions; thus, we do not need detailed information about the stimuli geometry and will only add the annotation for the number of dots presented on the screen. We provide a code snippet demonstrating the implementation of this time annotation. In this code snippet, we will load the annotation table S1 from a CSV file, but it is also possible to create the annotation fully with the code, as demonstrated in the Documentation under Quick Start: Adding information about the Time Annotation. Please note that the duration of stimuli can be provided in various units such as frames, seconds, volumes, etc. However, if units other than frames are used, it is required to provide a method for converting them to frames. In our example, we specify the stimulus duration in volumes and use the *timing_conversion* argument to indicate how volumes can be converted to frames.

```
# Load the experiment from the saved database
experiment = vx.Experiment.load("experiment.db")

# Load the CSV file with the numerosity annotation
numerosity_df = pd.read_csv("numerosity_annotation.csv", index_col = False)

# Add the time annotation to the experiment
# The timing_conversion specifies that
# 1 volume corresponds to frames_per_volume number of frames (67)
# The frames_per_volume is obtained from the experiment metadata
experiment.add_annotations_from_df(numerosity_df,
                                   cycles = True,
                                   timing_conversion= {"volumes":1, "frames":experiment.frames_per_volume})

# Save the changes to the experiment
experiment.save("experiment.db")
```

[2] Full link: https://github.com/LemonJust/numan/blob/main/notebooks/vodex_si/01_Define_experiment_and_create_dff_movie.ipynb

[3] Full link: https://github.com/LemonJust/numan/blob/main/notebooks/vodex_si/03_Extract_cell_signals.ipynb

duration_volumes	name	group	description
3	b	number	blank, no dots
1	d4	number	4 dots on the screen
9	b	number	blank, no dots
1	d3	number	3 dots on the screen
7	b	number	blank, no dots
1	d5	number	5 dots on the screen
5	b	number	blank, no dots
1	d2	number	2 dots on the screen
7	b	number	blank, no dots
1	d1	number	1 dot on the screen
9	b	number	blank, no dots
1	d4	number	4 dots on the screen
5	b	number	blank, no dots
1	d2	number	2 dots on the screen
7	b	number	blank, no dots
1	d5	number	5 dots on the screen
7	b	number	blank, no dots
1	d3	number	3 dots on the screen
5	b	number	blank, no dots
1	d1	number	1 dot on the screen
9	b	number	blank, no dots
1	d4	number	4 dots on the screen
9	b	number	blank, no dots
1	d1	number	1 dot on the screen
5	b	number	blank, no dots
1	d3	number	3 dots on the screen
9	b	number	blank, no dots
1	d2	number	2 dots on the screen
7	b	number	blank, no dots
1	d5	number	5 dots on the screen
6	b	number	blank, no dots

Table S1. Annotation for the Number of Dots Presented on the Screen

With VoDEx, loading the brain volumes recorded during the presentation of visual stimuli becomes effortless. We showcase a code snippet that highlights the simplicity of retrieving the volumes that correspond to each numerosity from the dataset.

```
# Get the indices of the volumes to load for each number of dots
d1_idx = experiment.choose_volumes(("number","d1")) # 1 dot
d2_idx = experiment.choose_volumes(("number","d2")) # 2 dots
d3_idx = experiment.choose_volumes(("number","d3")) # 3 dots
d4_idx = experiment.choose_volumes(("number","d4")) # 4 dots
d5_idx = experiment.choose_volumes(("number","d5")) # 5 dots

# Load the volumes for each number of dots
d1_volumes = experiment.load_volumes(d1_idx)
d2_volumes = experiment.load_volumes(d2_idx)
d3_volumes = experiment.load_volumes(d3_idx)
d4_volumes = experiment.load_volumes(d4_idx)
d5_volumes = experiment.load_volumes(d5_idx)
```

In addition to the specific-stimulus brain volumes, we also need to load the brain volumes captured during the blank period immediately preceding the stimuli. This allows us to isolate the changes in brain activity specifically due to stimuli presentation. VoDEx provides an intuitive method for extracting these blank volumes, and we provide a code snippet showcasing the ease of obtaining them.

```
# as blank, we will take the volumes immediately before the signals
b1_idx = [idx-1 for idx in d1_idx]
b2_idx = [idx-1 for idx in d2_idx]
b3_idx = [idx-1 for idx in d3_idx]
b4_idx = [idx-1 for idx in d4_idx]
b5_idx = [idx-1 for idx in d5_idx]
```

```

# load volumes corresponding to blank
b1_volumes = experiment.load_volumes(b1_idx)
b2_volumes = experiment.load_volumes(b2_idx)
b3_volumes = experiment.load_volumes(b3_idx)
b4_volumes = experiment.load_volumes(b4_idx)
b5_volumes = experiment.load_volumes(b5_idx)
```

With both the stimulus and blank brain volumes loaded, researchers can perform various data analysis tasks. We provide a Jupyter Notebook [4] that demonstrates the creation of the Statistical Parametric Map (SPM) using VoDEx. The SPM highlights the voxels in the brain images that exhibit significant responses to the stimuli.

2.3. Tracking Complex Visual Stimuli and Analyzing Cell Responses with VoDEx

Building upon the numerosity annotation introduced in Section 2.2, we extend our annotations to account for other non-numerical variables, allowing us to explore their influence on cell responses. To illustrate the implementation of this expanded time annotation, we provide a code snippet below. In this snippet, we add two separate annotations: a dot position and an individual dot size. As before, we will load the annotation from a CSV file. Please note that we can combine multiple annotations in one file, as shown below (Table S2).

duration_volumes	name	group	description
124	ch	position	equivalent convex hull
124	id	position	equivalent inter-distance
4	cr	size	constant radius
10	cr	size	constant radius
8	cr	size	constant radius
6	cr	size	constant radius
8	cr	size	constant radius
10	ta	size	equivalent total area
6	ta	size	equivalent total area
8	ta	size	equivalent total area
8	ta	size	equivalent total area
6	ta	size	equivalent total area
10	tp	size	equivalent total perimeter
10	tp	size	equivalent total perimeter
6	tp	size	equivalent total perimeter
10	tp	size	equivalent total perimeter
14	tp	size	equivalent total perimeter

Table S2. Annotation for Geometric Parameters of Visual Stimuli

```
# Load the experiment from the saved database
experiment = vx.Experiment.load("experiment.db")

# Load the CSV file containing the spread and size annotations
covariate_df = pd.read_csv("covariate_annotation.csv", index_col = False)

# Add the time annotation to the experiment
experiment.add_annotations_from_df(covariate_df,
                                   cycles = True,
                                   timing_conversion= {"volumes":1, "frames": experiment.frames_per_volume})

# Save the changes to the experiment database
experiment.save("experiment.db")
```

Analyzing cell responses in the context of complex visual stimuli involves comparing activity patterns across different sets of stimuli parameters. Since each timestep of the extracted cell signals corresponds to one volume for each cell, we can leverage VoDEx's querying capabilities to retrieve cell signals associated with specific combinations of numerical and non-numerical variables, as demonstrated below.

```
# Retrieve cell signal corresponding to the 5 dots with a constant radius
# (16 such timepoints)
```

[4] Full link: https://github.com/LemonJust/numan/blob/main/notebooks/vodex_si/02_Create_t-score_and diff_images.ipynb

```python
d5_cr_idx = experiment.choose_volumes([("number", "d5"),("size", "cr")])
# cell_signal: numpy array of shape (time_steps, )
# representing the cell signal previously extracted from the volumetric movie
d5_cr_signal = cell_signal[d5_cr_idx ]

# Retrieve cell signal corresponding to the 5 dots, a constant radius, and equivalent convex hull
# (8 such timepoints)
d5_ch_idx = experiment.choose_volumes([("number", "d5"),("size", "cr"),("position", "ch")])
d5_cr_ch_signal = cell_signal[d5_ch_idx]
```

With the ability to load the necessary signals, researchers can perform various data analysis tasks or organize the cell signals into a table for subsequent analysis in external software, as demonstrated below:

```python
# Retrieve all time points (volumes) during stimulus presentation for numerosities 1 to 5
stim_volumes = experiment.choose_volumes([("number", "d1"), ("number", "d2"), ("number", "d3"),
                                          ("number", "d4"), ("number", "d5")], logic="or")
# Get cell signal for these time points
stim_signal = cell_signal[stim_volumes]

# Get annotation information for these time points
annotation_dict = experiment.get_volume_annotations(stim_volumes)

# Create a summary dataframe combining cell signals and annotations
annotation_dict['cell_signal'] = stim_signal
annotation_df = pd.DataFrame(annotation_dict)

# Save the annotation dataframe to a CSV file
annotation_df.to_csv("stim_full_annotation.csv", index=False)
```

In this code snippet, we first retrieve the time points during stimulus presentation for numerosities 1 to 5. We then extract the corresponding cell signals and obtain the annotation information for these time points using VoDEx. Finally, we combine the cell signals and annotations into a summary dataframe and save it as a CSV file (Table S3). With the created annotation dataframe, one can perform various statistical analyses, including multi-factor ANOVA, to examine the effects of different factors on cell responses.

number	size	position	volumes	cell_signal
d4	cr	ch	3	0.222
d3	cr	ch	13	0.286
d5	cr	ch	21	0.068
...	...	...	...	...
...	...	...	...	...
d3	tp	id	1959	0.130
d2	tp	id	1969	0.265
d5	tp	id	1977	0.077

Table S3. Summary of Cell Signals and Annotations for Stimulus Presentation

Additionally, we provide a link to Jupyter Notebook Jupyter Notebook[5] that showcases how VoDEx simplifies the creation of plots representing neural activity in response to numerosity under different geometric constraints of the visual stimuli.

VoDEx's versatility as a tool for both data loading and analysis proves invaluable in uncovering the intricate neural cellular responses to visual numerosity stimuli.

References

H. Dana, Y. Sun, B. Mohar, B. K. Hulse, A. M. Kerlin, J. P. Hasseman, G. Tsegaye, A. Tsang, A. Wong, R. Patel, J. J. Macklin, Y. Chen, A. Konnerth, V. Jayaraman, L. L. Looger, E. R. Schreiter, K. Svoboda, and D. S. Kim. High-performance calcium sensors for imaging activity in neuronal populations and microcompartments. *Nature Methods*, 16(7):649–657, June 2019. doi: 10.1038/s41592-019-0435-6. URL `https://doi.org/10.1038/s41592-019-0435-6`.

A. D. Edelstein, M. A. Tsuchida, N. Amodaj, H. Pinkard, R. D. Vale, and N. Stuurman. Advanced methods of microscope control using manager software. *Journal of Biological Methods*, 1(2):e10, Nov. 2014. doi: 10.14440/jbm.2014.36. URL `https://doi.org/10.14440/jbm.2014.36`.

[5] Full link: https://github.com/LemonJust/numan/blob/main/notebooks/vodex_si/07_Plot_individual_covariates.ipynb

T. Gebuis, R. C. Kadosh, and W. Gevers. Sensory-integration system rather than approximate number system underlies numerosity processing: A critical review. *Acta Psychologica*, 171:17–35, Nov. 2016. doi: 10.1016/j.actpsy.2016.09.003. URL `https://doi.org/10.1016/j.actpsy.2016.09.003`.

K. Keomanee-Dizon, S. E. Fraser, and T. V. Truong. A versatile, multi-laser twin-microscope system for light-sheet imaging. *Review of Scientific Instruments*, 91(5):053703, May 2020. doi: 10.1063/1.5144487. URL `https://doi.org/10.1063/1.5144487`.

A. Messina, D. Potrich, M. Perrino, E. Sheardown, M. E. M. Petrazzini, P. Luu, A. Nadtochiy, T. V. Truong, V. A. Sovrano, S. E. Fraser, C. H. Brennan, and G. Vallortigara. Quantity as a fish views it: Behavior and neurobiology. *Frontiers in Neuroanatomy*, 16, July 2022. doi: 10.3389/fnana.2022.943504. URL `https://doi.org/10.3389/fnana.2022.943504`.

A. Nieder. The adaptive value of numerical competence. *Trends in Ecology &amp Evolution*, 35(7):605–617, July 2020. doi: 10.1016/j.tree.2020.02.009. URL `https://doi.org/10.1016/j.tree.2020.02.009`.

I. M. L. Pennock, T. T. Schmidt, D. Zorbek, and F. Blankenburg. Representation of visual numerosity information during working memory in humans: An scpfMRI/scp decoding study. *Human Brain Mapping*, 42(9):2778–2789, Mar. 2021. doi: 10.1002/hbm.25402. URL `https://doi.org/10.1002/hbm.25402`.

M. Piazza, V. Izard, P. Pinel, D. L. Bihan, and S. Dehaene. Tuning curves for approximate numerosity in the human intraparietal sulcus. *Neuron*, 44(3):547–555, Oct. 2004. doi: 10.1016/j.neuron.2004.10.014. URL `https://doi.org/10.1016/j.neuron.2004.10.014`.

M. Zanon, D. Potrich, M. Bortot, and G. Vallortigara. Towards a standardization of non-symbolic numerical experiments: GeNEsIS, a flexible and user-friendly tool to generate controlled stimuli. *Behavior Research Methods*, 54(1):146–157, June 2021. doi: 10.3758/s13428-021-01580-y. URL `https://doi.org/10.3758/s13428-021-01580-y`.
